# Association between circadian syndrome and the prevalence of kidney stones in overweight adults: a cross-sectional analysis of NHANES 2007–2018

**DOI:** 10.1186/s12889-023-15934-y

**Published:** 2023-05-26

**Authors:** Yunfei Xiao, Shan Yin, Yunjin Bai, Zhenzhen Yang, Jiahao Wang, Jianwei Cui, Jia Wang

**Affiliations:** 1 Department of Urology, Institute of Urology, West China Hospital, Sichuan University, No. 37, Guoxue Alley, Chengdu, Sichuan P.R. China; 2 Department of Urology, Affiliated Hospital of North Sichuan Medical College, Nanchong, China; 3 Department of Clinical Laboratory, Nanchong Central Hospital, Nanchong, China

**Keywords:** Circadian syndrome, Overweight, Kidney stones, Association, NHANES

## Abstract

**Objective:**

To explore the association between circadian syndrome (CircS) and the prevalence of kidney stones in overweight people.

**Materials and methods:**

A cross-sectional analysis was conducted based on the NHANES 2007–2018. Overweight people aged ≥ 20 years were the target population. Three multivariable logistic regression models were built to examine the association between CircS and kidney stones. Subgroup analysis based on age, gender, and race were also employed. Interaction and stratification analysis was also conducted to identify whether some factors modify the association.

**Result:**

A total of 4,603 overweight participants were included in the study. The multivariable logistic regression suggested that CircS was positively associated with the prevalence of kidney stones (OR = 1.422, 95% CI 1.057 to 1.912). The subgroup analysis showed that the association was more obvious in females (OR = 1.604, 95% CI 1.023 to 2.516) or in the population aged 35 to 49 years old (OR = 2.739, 95% CI 1.428 to 5.254). Additionally, the same trend was present when people were Mexican American (OR = 3.834, 95% CI 1.790 to 8.215) or other races (OR = 4.925, 95% CI 1.776 to 13.656). The interaction and stratification analysis showed that the results above were robust.

**Conclusion:**

CircS was positively associated with the prevalence of kidney stones in overweight people, especially people as females, aged 35 to 49, and Mexican Americans.

**Supplementary Information:**

The online version contains supplementary material available at 10.1186/s12889-023-15934-y.

## Introduction

Over the past 30 years, kidney stones have emerged as one of the most prevalent diseases, causing a major threat to human health globally. Studies have indicated that the prevalence of kidney stones is more common than ever before regardless of age, gender, or race [1]. Additionally, kidney stones can be treated well by numerous treatment modalities, but up to 50% of patients easily recur more than other urological diseases within 5 to 10 years after the first onset. The staggering healthcare expenditures exceeded US$2 billion annually in the United States [2]. These highlight the urgent need for improved preventive measures and cost-effective treatments. However, despite extensive research, the specific mechanisms of kidney stones remain unknown.

Through various studies, it has been discovered that there is a correlation between metabolic syndrome (MetS) and kidney stones [3]. Additionally, previous surveys have highlighted that both short sleep duration and depression contribute to the prevalence of urolithiasis, and are often accompanied by MetS [4, 5]. Nonetheless, those factors are always analyzed separately and never considered at the same time. With the introduction of the concept of circadian syndrome (CircS), this phenomenon seems to be scientifically explained. CircS is now considered an important underlying factor for MetS and its associated health issues [6]. The diagnosis of CircS requires the presence of at least four chronic disorders, including hypertension, dyslipidemia, waist circumference, diabetes, short sleep duration, and depression [7]. These components mentioned above are intricately regulated by the circadian rhythm, which plays a crucial role in regulating various aspects of human health and metabolism. Recent studies have shown that CircS may be a stronger predictor of cardiovascular diseases, lower urinary tract symptoms, and stroke compared to MetS. This highlights the potential for CircS to increase the risk of kidney stones [8–10]. In addition, modern lifestyles characterized by shift work, high-consumption diets, and less physical activity have been found to make the risk of circadian dysfunction increase, which further supports the hypothesis that CircS may be a relevant risk factor for kidney stones.

This study focuses on individuals who are overweight, as determined by their body mass index (BMI). Specifically, we are including those who fall within the overweight range, which is defined as having a BMI of 25 to less than 30 kg/m2. While BMI is a commonly used method for assessing weight status, it has been found to have poor specificity and sensitivity in diagnosing overweight individuals. Previous studies have shown that overweight individuals are more likely to have a higher percentage of body fat and struggle with accurately identifying their weight status, potentially leading to obesity [11]. In addition, overweight people have accounted for over one-third of the total population [12]. Given the high risk of CircS and kidney stones, a study focusing on overweight people is highly valuable for both research and clinical applications.

To the best of our knowledge, there is little research on elucidating the association between the prevalence of CircS and kidney stones in overweight adults. In this study, a cross-sectional study has been conducted to answer the question by analyzing large population data from the National Health and Nutrition Examination Survey (NHANES).

## Materials and method

### Study population

**Fig. 1:**
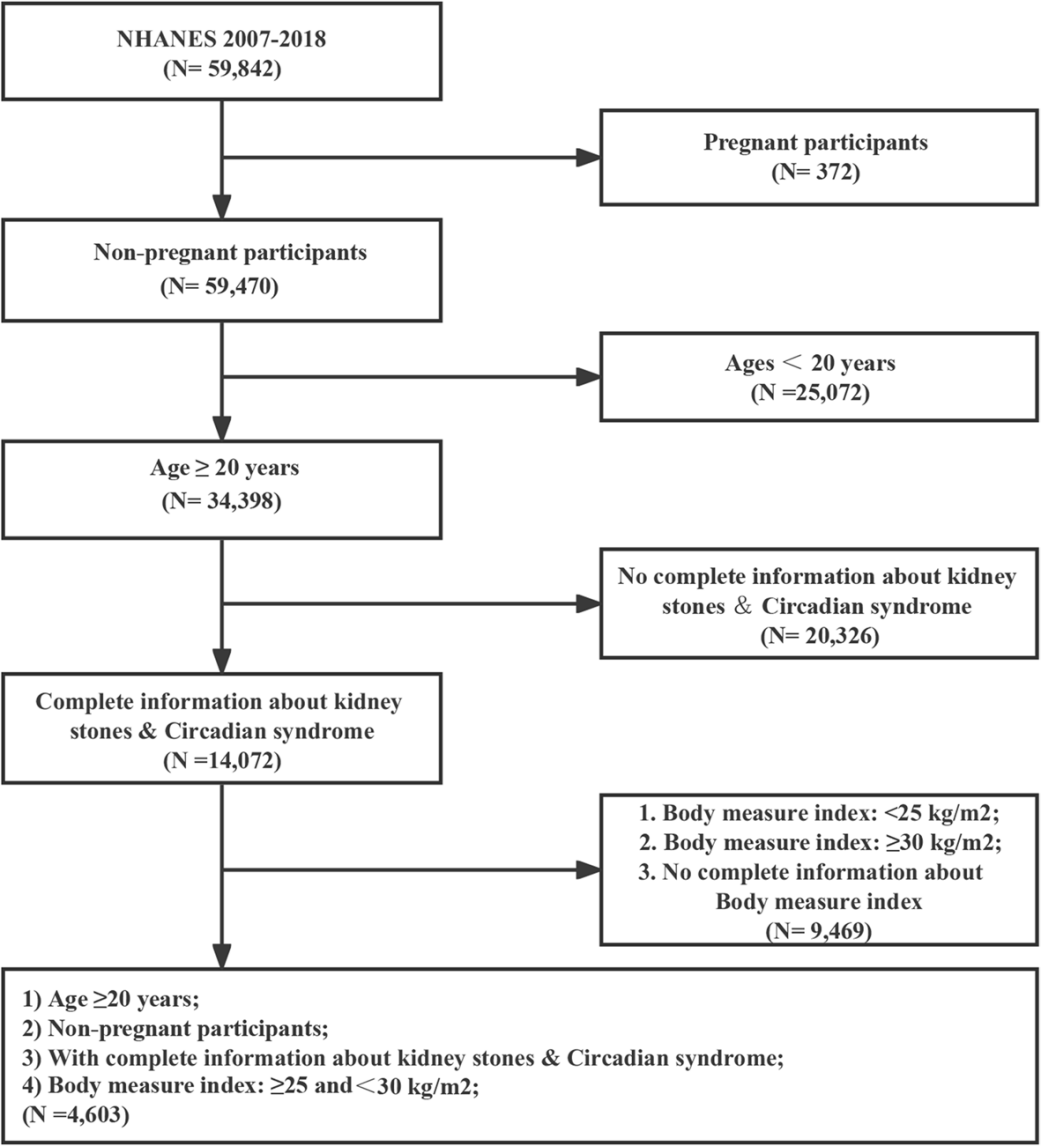
Flow diagram of obtaining the final inclusion in the population

NHANES is a survey that measures the health and nutritional status of Americans. Several stages of probability sampling are employed to gather information from a variety of sources, including interviews, physical exams, and lab tests. All data is cited from NHANES (https://www.cdc.gov/nchs/nhanes/index.htm). Researchers have access to this database for free since 1999, and it is updated regularly every two years. There were 59,842 participants involved in this study, with six cycles of NHANES (2007–2008, 2009–2010, 2011–2012, 2013–2014, 2015–2016, 2017–2018) cited. First, 372 pregnancies were excluded. In addition, participants under 20 years old (*n* = 25,072) were removed. Moreover, people were also excluded based on an incomplete kidney stone questionnaire or missing data for CircS diagnosis (*n* = 20,326). Furthermore, people with BMI < 25 kg/m^2^, ≥ 30 kg/m^2^, or missing information about it were not involved (*n* = 9469). Finally, a total of 4,603 participants were admitted **(**Fig. 1**)**.

All NHANES study protocols were approved by the Ethics Review Committee of the National Center for Health Statistics (NCHS), and consent was obtained from all participants.

### Outcomes and exposure variable

The prevalence of kidney stones was the major outcome of this study. Data from the questionnaire can be extracted to calculate the prevalence of kidney stones. In the questionnaire, the participants with kidney stones developed were defined by their answer “yes” to the question “Have you ever had kidney stones?”, and those who responded “no” were assumed not to suffer from kidney stones.

The primary exposure variable was CircS, and the diagnosis of CircS was based on the following components. People with ≥ 4 of the following components were defined as a circadian syndrome [9]: (1) women with a waist circumference of ≥ 88 cm and men with a waist circumference of ≥ 102 cm; (2) elevated triglycerides (≥ 150 mg/dL) or use of lipid-lowering medication; (3) decreased high-density lipoprotein-cholesterol (< 40 mg/dL in men and < 50 mg/dL in women) or use of lipid-lowering medication; (4) elevated blood pressure (systolic ≥ 130 or diastolic ≥ 85 mmHg) or use of an antihypertensive drug; (5) elevated fasting glucose (≥ 100 mg/dL) or use of anti-diabetic medication; (6) reduced sleep duration (≤ 6 h/day); (7) depression symptoms defined by the patient health status questionnaire-9.

### Covariates’ collection

From the questionnaires, the baseline information regarding renal calculi and CircS was obtained in large part. The continuous variables included age (≥ 20 years), poverty income ratio (PIR), BMI, energy, and Healthy Eating Index (HEI)-2015 [13]. Categorical variables included gender, age, race, education level, marital status, PIR, smoking, alcohol, cancer, gout, stroke, vigorous activity, and moderate activity (all no/yes). Specifically, the ages of participants were stratified as follows: 20–34, 35–49, 50–64, and ≥ 65 years. PIR was grouped as ≤ 1.3, > 1.3 & ≤ 3.5, > 3.5%, and missing. Races included Mexican American, Other Hispanic, non-Hispanic black, non-Hispanic white, and other races. Smoking included never (< 100 cigarettes in life), former (≥ 100 cigarettes in life and smoking not at all now), and now (≥ 100 cigarettes in life and smoking some days or every day in life). Alcohol was defined as < 12 or ≥ 12 drinks per year. Marital status was divided into married, widowed, divorced, separated, never married, and living with a partner. Education levels were classified into five categories as follows: less than 9th grade, 9th-11th grade, high school graduate, some college, and college graduate or above. There were missing data for both PIR and alcohol.

### Statistical analysis

For all analyses, sampling weights recommended by the Centers for Disease Control and Prevention (CDC) were used. To describe the baseline characteristics of all participants, the mean ± standard deviation (SD) and frequency (proportions) were fully used. Specifically, the correlation of continuous variables was conducted using linear regression, while categorical variables were compared using chi-square analysis.

To assess the relationship between CircS and kidney stones more effectively, a total of two groups were formed based on CircS diagnosis, and three logistic regression models were built to analyze the correlation between CircS and kidney stones. In the non-adjusted model, no factor was adjusted. The minimally-adjusted model was only adjusted for gender, age, and race. The fully-adjusted model was further adjusted for education, BMI, marital status, PIR, smoking, alcohol, cancer, gout, stroke, vigorous activity, moderate activity, energy, and HEI-2015 total score. Given the observed difference in descriptive characteristics in previous studies, the possible confounding covariates (age, gender, and race included) were evaluated by subgroup analysis in three logistic regression models. Additionally, to explore the influence of covariates on the association between CircS and urolithiasis, sensitivity analysis was conducted through the incorporation of the interaction and stratification term into regression models.

Statistical analyses were all performed using R 4.0 (http://www.R-project.org, the R Foundation) and EmpowerStats (http://www.empowerstats.com, X&Y Solutions, Inc.). Differences were considered statistically significant when P was less than 0.05.

## Result

### Baseline characteristics of study participants

From the NHANES 2007–2018 cycle, a total of 4,603 participants were eligible, and specific inclusion and exclusion criteria were shown in Fig. 1. Most of the baseline characteristics differed statistically significantly among the groups divided by the CircS. As the table (Table 1) showed, people with CircS were older (mean ± SD = 61.65 ± 13.79 years), and the age of those participants mainly varied from 50 to more. Moreover, the prevalence of kidney stones was significantly higher in this group (14.50%). Additionally, compared to non-CircS, the patients were more likely to be female (45.93%), had a slightly higher BMI (27.66 ± 1.38 kg/m^2^) and HEI-2015 (51.92 ± 12.98), and a higher proportion of gout (7.49%), cancer (18.94%), and stroke (6.40%). However, energy intake (2005.12 ± 893.55 kcal) and PIR (2.94 ± 1.64), the proportion of alcohol (61.03%), non-smoking (never smoking) (46.86%), moderate activity (39.12%), and high education (> high school graduate) (51.96%) were lower than non-CircS participants. Unfortunately, no significant difference in vigorous activities between the two groups was found. In addition, a higher prevalence of CircS was present in people with kidney stones (Table 2).

**Table 1:** Characteristics of participants by categories of circadian syndrome: NHANES 2007-2018^a^

Variables	All (*n* = 4,603)	Groups		*P*-value
		**Non-circadian syndrome (*n* = 3,044)**	**Circadian syndrome (*n* = 1,559)**	
Age (years, mean ± SD)	50.64 ± 16.62	46.30 ± 15.60	61.65 ± 13.79	< 0.001
20–34 (%)	20.21	26.74	3.64	
35–49 (%)	27.56	31.91	16.52	
50–64 (%)	28.61	26.50	33.98	
≥ 65 (%)	23.61	14.85	45.86	
PIR (mean ± SD)	3.09 ± 1.64	3.14 ± 1.63	2.94 ± 1.64	< 0.001
≤ 1.3 (%)	19.11	18.54	20.56	
> 1.3 and ≤ 3.5 (%)	32.57	31.79	34.54	
> 3.5 (%)	41.71	43.58	36.99	
Missing (%)	6.61	6.09	7.92	
BMI (kg/m^2^, mean ± SD)	27.42 ± 1.40	27.32 ± 1.39	27.66 ± 1.38	< 0.001
HEI-2015 (mean ± SD)	51.26 ± 13.60	51.01 ± 13.83	51.92 ± 12.98	0.044
Energy (kcal, mean ± SD)	2181.62 ± 947.31	2250.03 ± 958.65	2005.12 ± 893.55	< 0.001
Kidney stones (%)				< 0.001
No	89.80	91.50	85.50	
Yes	10.02	8.50	14.50	
Gender (%)				0.001
Male	57.79	59.26	54.07	
Female	42.21	40.74	45.93	
Education (%)				< 0.001
Less than 9th grade	6.31	5.38	8.67	
9-11th grade	10.05	8.80	13.21	
High school graduate	22.78	21.46	26.16	
Some college	29.80	30.19	28.79	
College graduate or above	31.06	34.17	23.17	
Race (%)				< 0.001
Mexican American	9.37	10.41	6.72	
Other Hispanic	6.58	6.93	5.69	
Non-Hispanic white	68.42	67.13	71.71	
Non-Hispanic black	8.85	9.07	8.29	
Other races	6.78	6.45	7.59	
Marital (%)				< 0.001
Married	59.25	58.36	61.52	
Widowed	6.27	3.74	12.71	
Divorced	10.71	9.96	12.61	
Separated	1.94	1.98	1.85	
Never married	13.85	16.85	6.23	
Living with partner	7.97	9.11	5.08	
Gout (%)				< 0.001
No	96.24	97.71	92.51	
Yes	3.76	2.29	7.49	
Cancer (%)				< 0.001
No	89.06	92.21	81.06	
Yes	10.94	7.79	18.94	
Smoking (%)				< 0.001
Never	53.19	55.68	46.86	
Former	29.12	26.78	35.05	
Now	17.69	17.53	18.09	
Alcohol (%)				< 0.001
No	16.84	15.13	21.17	
Yes	66.94	69.26	61.03	
Missing	16.22	15.60	17.80	
Stroke (%)				< 0.001
No	96.98	98.32	93.60	
Yes	3.02	1.68	6.40	
Vigorous activity (%)				0.076
No	77.05	76.36	78.80	
Yes	22.95	23.64	21.20	
Moderate activity (%)				0.010
No	57.88	56.69	60.88	
Yes	42.12	43.31	39.12	

Values are expressed as mean + SD or %. *SD* Standard deviation, *BMI* Body mass index, *PIR* Poverty income ratio, *HEI-2015* Healthy eating index-2015

^a^For continuous variables, the t-test for slope was used in generalized linear models

**Table 2:** Characteristics of 4,603 participants in NHANES between 2007 and 2018^a^

Variables	All (*n* = 4,603)	Groupe		*P*-value
		**Non-kidney stone (*n* = 4,125))**	**Kidney stones (*n* = 478)**	
Age (years, mean ± SD)	50.64 ± 16.62	50.04 ± 16.58	55.84 ± 16.02	< 0.001
20–34 (%)	20.21	21.19	11.62	
35–49 (%)	27.56	28.07	23.12	
50–64 (%)	28.61	28.08	33.33	
≥ 65 (%)	23.61	22.66	31.93	
PIR (mean ± SD)	3.09 ± 1.64	3.09 ± 1.65	3.05 ± 1.57	0.590
≤ 1.3 (%)	19.11	19.39	16.63	
> 1.3 and ≤ 3.5 (%)	32.57	32.09	36.78	
> 3.5 (%)	41.71	42.07	38.60	
Missing (%)	6.61	6.45	7.99	
BMI (kg/m^2^, mean ± SD)	27.42 ± 1.40	27.41 ± 1.40	27.53 ± 1.41	0.068
HEI-2015 (mean ± SD)	51.26 ± 13.60	51.38 ± 13.60	50.26 ± 13.60	0.096
Energy (kcal, mean ± SD)	2181.62 ± 947.31	2184.21 ± 955.66	2158.82 ± 870.05	0.588
Circadian syndrome (%)				< 0.001
No	71.75	73.10	59.82	
Yes	28.25	26.90	40.18	
Gender (%)				< 0.001
Male	57.79	56.91	65.59	
Female	42.21	43.09	34.41	
Education (%)				0.006
Less than 9th grade	6.31	6.29	6.50	
9-11th grade	10.05	10.09	9.72	
High school graduate	22.78	23.09	20.09	
Some college	29.80	28.97	37.06	
College graduate or above	31.06	31.56	26.64	
Race (%)				< 0.001
Mexican American	9.37	9.54	7.88	
Other Hispanic	6.58	6.48	7.41	
Non-Hispanic white	68.42	67.64	75.32	
Non-Hispanic black	8.85	9.35	4.43	
Other races	6.78	6.98	4.96	
Marital (%)				< 0.001
Married	59.52	58.15	68.99	
Widowed	6.27	6.31	5.91	
Divorced	10.71	10.78	10.09	
Separated	1.94	1.86	2.68	
Never married	13.85	14.61	7.19	
Living with partner	7.97	8.30	5.13	
Gout (%)				0.028
No	96.24	96.45	94.40	
Yes	3.76	3.55	5.60	
Cancer (%)				< 0.001
No	89.06	90.03	80.56	
Yes	10.94	9.97	19.44	
Smoking (%)				0.327
Never	53.19	53.56	50.00	
Former	29.12	28.84	31.55	
Now	17.69	17.61	18.44	
Alcohol (%)				0.974
No	16.84	16.80	17.22	
Yes	66.94	66.97	66.64	
Missing	16.22	16.23	16.15	
Stroke (%)				0.432
No	96.98	97.05	96.40	
Yes	3.02	2.95	3.60	
Vigorous activity (%)				< 0.001
No	77.05	77.76	70.66	
Yes	22.95	22.22	29.34	
Moderate activity (%)				0.582
No	57.88	58.01	56.69	
Yes	42.12	41.99	43.31	

Values are expressed as mean + SD or %. *SD* Standard deviation, *BMI* Body mass index, *PIR* Poverty income ratio, *HEI-2015* Healthy eating index-2015

^a^For continuous variables, the t-test for slope was used in generalized linear models

### Multivariate regression analysis

It was evident from the multivariate regression analysis that there was a positive association between the prevalence of nephrolithiasis and the prevalence of CircS in the non-adjusted model (OR = 1.826, 95% CI 1.449 to 2.300, *P* < 0.001), the minimally adjusted model (OR = 1.523, 95% CI 1.184 to 0.959, *P* < 0.001) and the fully adjusted model (OR = 1.422, 95% CI 1.057 to 1.912, *P* = 0.021) (Table 3).

**Table 3:** Association of circadian syndrome with the prevalence of kidney stones

Variables	Non-adjusted model^a^		Minimally adjusted model^b^		Fully adjusted model^c^	
	**OR (95%CI)**	**P**	**OR (95%CI)**	**P**	**OR (95%CI)**	**P**
**Circadian syndrome**						
No	Ref		Ref		Ref	
Yes	1.826 (1.449, 2.300)	< 0.001	1.523 (1.184, 1.959)	< 0.001	1.422 (1.057, 1.912)	0.021

CI: confidence interval, OR: odds ratio

^a^Non-adjusted model adjusts for none

^b^ Minimally adjusted model adjusts for age, gender, race

^c^ Fully adjusted model adjusts for age, gender, education, race, BMI, marital, PIR, gout, cancer, energy, HEI-2015, smoking, vigorous activity, moderate activity, alcohol, stroke

### Multivariate regression analysis based on age, gender, and race

In the subgroup analysis based on age, we observed that the risk of kidney stones was 2.109 times higher when people with CircS were aged only from 35 to 49 years in the fully-adjusted model (OR = 2.739, 95% CI 1.428 to 5.254, *P* = 0.004) (Table 4). There was no more significant association between CircS and renal calculi in other ages. In addition, females with CircS preferred to develop renal stones than non-CircS people in the fully-adjusted model (OR = 1.604, 95% CI 1.023 to 2.516, *P* = 0.044). Moreover, we also conducted a further study on the correlation between kidney stones and race. As the results showed, when participants with CircS were Mexican American (OR = 3.834, 95% CI 1.790 to 8.215, *P* = 0.001) or other races (non-Hispanic white, non-Hispanic black and other Hispanic excluded) (OR = 4.925, 95% CI 1.776 to 13.656, *P* = 0.004), it is worth noting that they were more likely to develop kidney stones in the fully-adjusted model.

**Table 4:** Association of circadian syndrome with the prevalence of kidney stones based on age, gender, and race

Variables	Circadian syndrome	Non-adjusted model^a^		Minimally adjusted model^b^		Fully adjusted model^c^	
		**OR (95%CI)**	**P**	**OR (95%CI)**	**P**	**OR (95%CI)**	**P**
**Age (years)**							
20–34							
	Circadian syndrome						
	No	Ref		Ref		Ref	
	Yes	0.159 (0.021,1.229)	0.081	0.152 (0.019,1.191)	0.076	0.938 (0.545, 1.615)	0.819
35–49							
	Circadian syndrome						
	No	Ref		Ref		Ref	
	Yes	1.550 (0.917,2.621)	0.105	1.587 (0.923,2.729)	0.098	**2.739 (1.428, 5.254)**	**0.004**
50–64							
	Circadian syndrome						
	No	Ref		Ref		Ref	
	Yes	1.694 (1.014,2.829)	0.047	1.739 (1.033,2.927)	0.040	1.329 (0.748, 2.361)	0.335
≥ 65							
	Circadian syndrome						
	No	Ref		Ref		Ref	
	Yes	1.425 (0.911,2.228)	0.124	1.451 (0.925,2.276)	0.108	1.756 (0.945, 3.263)	0.074
**Gender**							
Male							
	Circadian syndrome						
	No	Ref		Ref		Ref	
	Yes	2.021 (1.508,2.709)	< 0.001	1.358 (0.976,1.890)	0.073	1.236 (0.803, 1.904)	0.340
Female							
	Circadian syndrome						
	No	Ref		Ref		Ref	
	Yes	1.623 (1.028,2.564)	0.040	1.827 (1.149,2.904)	0.013	**1.604 (1.023, 2.516)**	**0.044**
**Race**							
Mexican American							
	Circadian syndrome						
	No	Ref		Ref		Ref	
	Yes	1.886 (0.981,3.626)	0.063	1.223 (0.627,2.387)	0.558	**3.834 (1.790, 8.215)**	**0.001**
Other Hispanic							
	Circadian syndrome						
	No	Ref		Ref		Ref	
	Yes	2.054 (1.153,3.658)	0.0178	2.594 (1.349,4.986)	0.006	0.757 (0.308, 1.856)	0.545
Non-Hispanic white							
	Circadian syndrome						
	No	Ref		Ref		Ref	
	Yes	1.771 (1.323,2.371)	< 0.001	1.474 (1.070,2.030)	0.020	1.195 (0.837, 1.706)	0.331
Non-Hispanic black							
	Circadian syndrome						
	No	Ref		Ref		Ref	
	Yes	2.550 (1.269,5.124)	0.011	1.657 (0.706,3.885)	0.251	0.535 (0.238, 1.205)	0.136
Other races							
	Circadian syndrome						
	No	Ref		Ref		Ref	
	Yes	1.539 (0.635,3.730)	0.344	1.740 (0.774,3.914)	0.186	**4.925 (1.776, 13.656)**	**0.004**

*CI* Confidence interval, *OR* Odds ratio

^a^Non-adjusted model adjusts for none

^b^ Minimally adjusted model adjusts for age, gender, race

^c^ Fully adjusted model adjusts for age, gender, education, race, BMI, marital, PIR, gout, cancer, energy, HEI-2015, smoking, vigorous activity, moderate activity, alcohol, stroke

### Stratification and interaction analysis

To determine whether some factors potentially affected the relationship between CircS and urolithiasis, an interaction analysis was performed (Table S1). As the results showed, we did not find any significant interaction, and no factor could modify the positive significant association between CircS and the prevalence of kidney stones. Namely, the statistically significant relationship between the two was robust and not influenced by these covariates.

## Discussion

This cross-sectional study aimed to evaluate the association between kidney stones and CircS in the overweight population, and the data were extracted and analyzed from 6 cycles of NHANES. The results demonstrated that overweight people with CircS were positively associated with the prevalence of kidney stones, especially females, people aged 35 to 49, Mexican American, and other races (non-Hispanic white, non-Hispanic black, and other Hispanic excluded). Moreover, the interaction and stratification analysis showed there was no factors modified the association. Namely, the results in this study were robust.

Several studies have shown that there is a link between MetS and kidney stones, regardless of the nature and size of the stone. Furthermore, individuals who suffer from sleep deprivation and depression are more likely to develop renal calculi and often exhibit a concomitant presence of MetS. Some evidence supports that disruptions in circadian rhythm and epigenetics may be key factors in explaining the risk determinants (MetS and its comorbidities). It has been demonstrated that all CircS components are associated with circadian disruption. For example, Goni et al. disclose that people without circadian rhythm-related MTNR1B gene are less marked for obesity improvement [14]. A multicenter study declares disorders in hormone secretion are easier to see in shift workers [15]. Moreover, Hoyos et al. report that circadian misalignments are evident in older people with depression [16]. Therefore, it is worth noting that this is the first work to explore the relationship between kidney stones and CircS, which is closely related to circadian disruption [17]. As expected, CircS is positively related to the prevalence of kidney stones in this work.

It is well known that CircS mainly consisted of circadian rhythm dysfunction, which is a set of biological variables produced by biological clocks (with approximately a 24-h period) [18]. Not only do the daily physiological and molecular rhythm are controlled, but the rhythmic expression of genes is also controlled by circadian clocks [19]. Thus, when dysfunctions develop, a variety of physiological and pathological disorders occur in the body, such as MetS, cardiovascular diseases, and neuropsychiatric disorders [20, 21]. All of these factors mentioned above can promote the development of kidney stones. Additionally, recent studies indicate that circadian rhythm mainly depended on a master “Body clock” regardless of peripheral clocks, which reside in the suprachiasmatic nucleus (SCN) of the hypothalamus [22]. The master “Body clock” is mainly affected by the influence of light intensity and duration [23]. Namely, incorrect exposure to light would contribute to CircS and kidney stone prevalence. Clinical studies reveal that shift workers always experience an altered circadian clock and bright light at night disrupted rhythm regularity, which accelerates chronic disorders development, including CVD, MetS, kidney stones, etc. [24, 25]. However, whether the duration and intensity of light affect kidney stones is still unclear. Apart from light, the master “Body clock” is also affected by numerous environmental factors and habits, including activities, diet, temperature, humidity, etc. [13, 26]. To make the invention to the development of kidney stones, make sure we keep a healthy lifestyle and pace of life involving regular schedules and healthy eating patterns urgently required.

Furthermore, as a critical role in metabolism, adiponectin has attracted much attention. CircS makes a contribution to the development of kidney stones through changes in adiponectin levels. Recurrent studies have shown that there is a bidirectional regulatory relationship between circadian rhythm and adiponectin [27]. The circadian clock (BMAL 1) mediates adiponectin expression through its transcription factor peroxisome proliferator-activated receptor-γ (PPARγ) and co-activator PGC-1α. Moreover, circadian rhythm disturbance and abnormal expression of BMAL1 are induced by adiponectin deficiency [28–30]. It is worth that overweight people and participants with short sleep down-regulate the content of adiponectin [31]. In addition to the antioxidant functions of normal biological rhythms, adiponectin does act as a preventive factor for kidney crystal formation through the antioxidant response, inhibition of apoptosis, and autophagy [32, 33]. Thus, adiponectin may play a central role in the association between CircS and kidney stones, and more basic research is required to make a deep exploration.

Aware of the gut microbiota's importance for endocrine metabolic disease and chronic disorders, recent evidence indicates that disruption of circadian rhythm modifies the gut microbiota, including the diversity and quantity of microbiome communities changed, host metabolism disturbed, and inflammatory reaction [34]. As reported, the gut microbiotas regulate not only oxalate absorption but also epithelial oxalate transport. Apart from that, oxalate metabolism is also governed by the microbiota or their metabolites. As circadian rhythm dysfunction occurred, the odds of hyperoxaluria, hyperoxaluria, and chronic oxalate nephropathy are raised. Owing to these variations, the process of calcium oxalate (the most common type of kidney stones) could be accelerated [35]. Unfortunately, the precise mechanisms behind the phenomenon are still dim, and the future work of our team will be guided by the question.

This study also revealed that race, gender, and age differed in the association between CircS and kidney stones among the overweight population. The association between the two became more obvious when overweight subjects were female. In addition to Mets being more common in females, the population prefers to develop hyperuricemia than males [36]. However, despite the differences narrowing, males are nearly twice as likely to develop kidney stones as females [9, 37]. So, the results need to be explained by some direct evidence. According to the subgroup analysis, people aged 35 to 49 made the positive relationship between renal calculi and CircS more significant. Although many lines of evidences indicate that the elderly account for the largest portion of CircS, numerous analyses support that these middle-aged populations are more easily to develop metabolism disorders and kidney stones in times of rapid change [38, 39]. Mounting evidence shows that the highest odds of kidney stones were seen for people aged 40 to 69 [40, 41], and these middle-aged people, especially overweight people, prefer to develop sleep disorders and psychological problems such as depression and anxiety [42]. Therefore, we should not only pay much attention to the mental and bodily health of children, adolescents, the young, and the elderly but also not overlook these middle-aged people. Additionally, differences in race were also present in our study. Compared to other racial groups, the positive correlations were stronger in all three models in Mexican America. The current evidence also proves that Mexican Americans were 1.6–1.7 times more likely to develop DM than non-Hispanic whites [43]. Furthermore, a higher ratio of poor overall health was seen among Mexican America than others, and Mexican America with MetS up-regulated the risk of poor overall health [44, 45]. In other words, poorer health status and a higher risk of kidney stones and CircS are consistent with Mexican Americans. Our previous study has found that diet plays an essential role in the formation of kidney stones [13]. Thus, to gain a more comprehensive understanding of racial differences, it is crucial to consider the prevalent dietary patterns in the US. The typical American diet is often characterized by a high intake of processed foods, refined sugar, and saturated fat, coupled with a low intake of whole grains, vegetables, and fruits. However, there are some differences in the traditional dietary habits of different races. Traditional diets of Mexican Americans are high in sodium from processed foods, high in animal protein, and low in calcium and water [46, 47]. Thus, lower dietary quality and higher risk of metabolic disorders are more pronounced in Mexican Americans [48]. These dietary factors, combined with genetic predisposition and lifestyle choices, could potentially contribute to an increased risk of kidney stone formation in the population. Considering these dietary influences and racial disparities, it is crucial to prioritize prevention and intervention strategies for kidney stones in Mexican Americans.

In this study, the merits and disadvantages both existed. First, this representative study of overweight populations in the United States includes a large number of participants based on NHANES. Second, CircS is a new concept combined with biological rhythms that are more consistent with the majority of physiological processes. To the best of our knowledge, no study has focused on the association between CircS and kidney stones until now. Third, the target population is overweight people who lacked full consciousness of body weight and health status, so the clinical application prospects are encouraging. In contrast, it cannot elaborate on the causality between CircS and kidney stone prevalence because of the cross-sectional design. Additionally, some information about the nature and size of kidney stones is not accessed from NHANES. Moreover, the duration of CircS is also non-existent. Thus, studies should be conducted in greater depth to validate the causal relationship between the prevalence of kidney stones and CircS.

## Conclusion

This study reveals that there is a positive relationship between CircS and kidney stones in overweight people. Moreover, the association becomes more obvious when the overweight participants are female, aged 35 to 49, and Mexican American. However, more high-quality prospective cohort studies are needed to explore the causal association between CircS and kidney stones.

## Supplementary Information

**Additional file 1:** **Table S1.** Logistic regression analysis to identify variables that modify the correlation between circadian syndrome and the prevalence of kidney stones.

## Data Availability

Data available in a publicly accessible repository that does not issue DOIs. Publicly available datasets were analyzed in this study. These data can be found here: https://www.cdc.gov/nchs/nhanes/index.htm.
