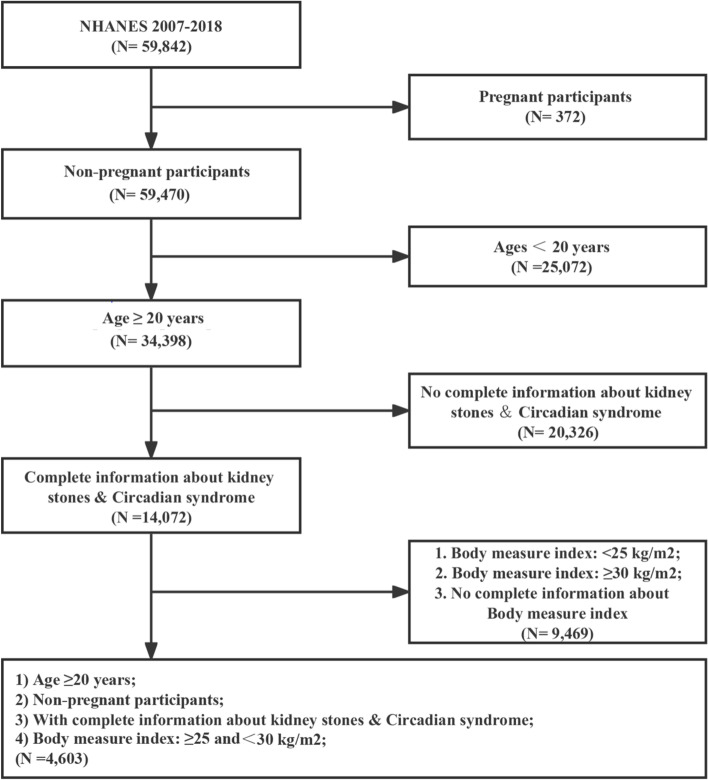# Correction: Association between circadian syndrome and the prevalence of kidney stones in overweight adults: a cross-sectional analysis of NHANES 2007–2018

**DOI:** 10.1186/s12889-023-16029-4

**Published:** 2023-06-12

**Authors:** Yunfei Xiao, Shan Yin, Yunjin Bai, Zhenzhen Yang, Jiahao Wang, Jianwei Cui, Jia Wang

**Affiliations:** 1 Department of Urology, Institute of Urology, West China Hospital, Sichuan University, No. 37, Guoxue Alley, Chengdu, Sichuan P.R. China; 2 Department of Urology, Affiliated Hospital of North Sichuan Medical College, Nanchong, China; 3 Department of Clinical Laboratory, Nanchong Central Hospital, Nanchong, China

**Correction: BMC Public Health 23, 960 (2023)**

**https://doi.org/10.1186/s12889-023-15934-y**

Following publication of the original article [1], the authors identified an error in the labels of Fig. 1. The incorrect and correct version of Fig. 1 are shown in in this correction article. The original article has been updated.

**Fig. 1:**
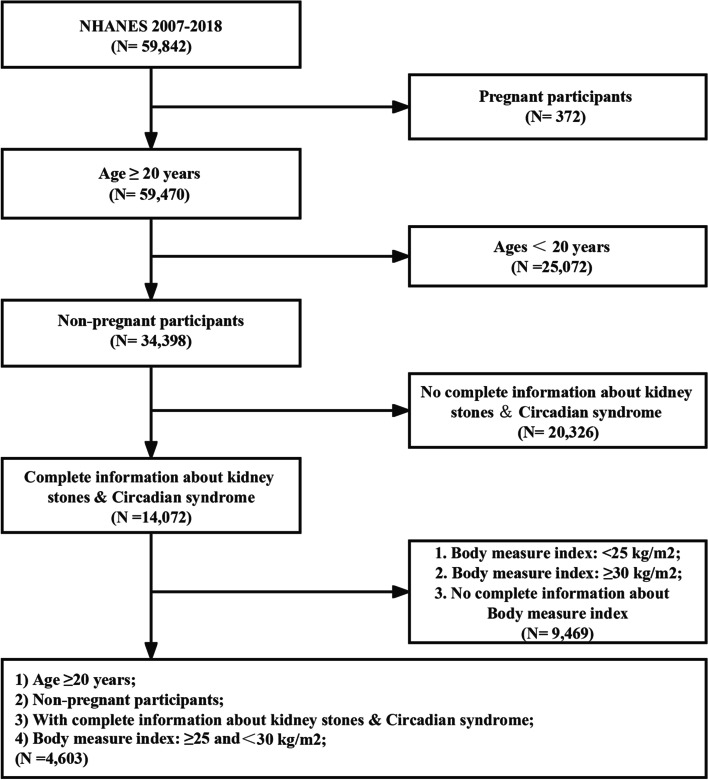
incorrect version of Fig. 1 as originally published

**Fig. 2** correct version of Fig. 1