# Supplementary material for: Association between circadian syndrome and the prevalence of kidney stones in overweight adults: a cross-sectional analysis of NHANES 2007–2018
Source: BMC Public Health. 2023 May 26;23:960. doi: 10.1186/s12889-023-15934-y (PMC10214633; doi:10.1186/s12889-023-15934-y)
Supplement: Supplementary file 1 — Additional file 1: Table S1. Logistic regression analysis to identify variables that modify the correlation between circadian syndrome and the prevalence of kidney stones. [file 12889_2023_15934_MOESM1_ESM.docx]

**Table S1** Logistic regression analysis to identify variables that modify the correlation between circadian syndrome and the prevalence of kidney stones.

| **Variables (%)** | **Non-adjusted model*** | | **Minimally adjusted model**** | | **Fully adjusted model***** | |
| --- | --- | --- | --- | --- | --- | --- |
|  | **OR (95%CI)** | **P for interaction** | **OR (95%CI)** | **P for interaction** | **OR (95%CI)** | **P for interaction** |
| Gender |  | 0.456 |  | 0.569 |  | 0.769 |
| Male | 2.021 (1.508, 2.709) |  | 1.540 (1.107, 2.142) |  | 1.278 (0.868, 1.882) |  |
| Female | 1.624(1.028, 2.564) |  | 1.298 (0.811, 2.078) |  | 1.167 (0.713, 1.909) |  |
| Age |  | 0.155 |  | 0.161 |  | 0.224 |
| 20-34 | 0.159 (0.021,1.229) |  | 0.165 (0.021,1.310) |  | 0.156 (0.020, 1.245) |  |
| 35-49 | 1.550 (0.917,2.621) |  | 1.583 (0.932,2.689) |  | 1.352 (0.783, 2.334) |  |
| 50-64 | 1.694 (1.014,2.829) |  | 1.735 (1.028,2.928) |  | 1.383 (0.748, 2.557) |  |
| ≥65 | 1.425 (0.911,2.228) |  | 1.444 (0.923,2.258) |  | 1.292 (0.779, 2.146) |  |
| Race |  | 0.866 |  | 0.881 |  | 0.664 |
| Mexican American | 1.886 (0.981,3.626) |  | 1.517 (0.777, 2.962) |  | 1.628 (0.787, 3.367) |  |
| Other Hispanic | 2.054 (1.153,3.658) |  | 1.659 (0.904, 3.044) |  | 1.350 (0.652, 2.797) |  |
| Non-Hispanic white | 1.771 (1.323,2.371) |  | 1.404 (1.026, 1.922) |  | 1.163 (0.811, 1.669) |  |
| Non-Hispanic black | 2.550 (1.269,5.124) |  | 2.024 (0.978, 4.190) |  | 2.042 (0.953, 4.378) |  |
| Other races | 1.539 (0.635,3.730) |  | 1.300 (0.524, 3.224 |  | 1.107 (0.367, 3.336) |  |
| Education |  | 0.595 |  | 0.690 |  | 0.361 |
| Less than 9th grade | 1.778 (0.872,3.626) |  | 1.457 (0.703, 3.020) |  | 1.120 (0.492, 2.550) |  |
| 9-11th grade | 1.388 (0.724,2.662) |  | 1.137 (0.568, 2.275) |  | 0.949 (0.463, 1.947) |  |
| High school graduate | 1.326 (0.718,2.451) |  | 1.043 (0.547, 1.986) |  | 0.787 (0.428, 1.446) |  |
| Some college | 2.010 (1.292,3.127) |  | 1.589(1.020, 2.475) |  | 1.349 (0.822, 2.215) |  |
| College graduate or above | 2.400 (1.428,4.034) |  | 1.804(1.051, 3.097) |  | 1.771 (1.004, 3.121) |  |
| Marital |  | 0.607 |  | 0.673 |  | 0.539 |
| Married | 1.624 (1.216,2.168) |  | 1.366 (1.005, 1.857) |  | 1.124 (0.770, 1.638) |  |
| Widowed | 1.789 (0.830,3.855) |  | 1.621 (0.748, 3.514) |  | 1.656 (0.685, 4.005) |  |
| Divorced | 1.491 (0.690,3.222) |  | 1.380 (0.627, 3.036) |  | 1.011 (0.498, 2.054) |  |
| Separated | 1.509 (0.447,5.098) |  | 1.392 (0.415, 4.664) |  | 1.391 (0.417, 4.640) |  |
| Never married | 4.712 (1.598,13.897) |  | 3.740 (1.307, 10.704) |  | 3.565 (1.135, 11.195) |  |
| Living with partner | 2.010 (0.659,6.134) |  | 1.498 (0.508, 4.418) |  | 1.264 (0.398, 4.014) |  |
| PIR |  | 0.039 |  | 0.034 |  | 0.056 |
| ≤1.3 | 1.578 (0.989,2.516) |  | 1.255 (0.769, 2.047) |  | 1.244 (0.751, 2.061) |  |
| >1.3 and ≤3.5 | 1.978 (1.319,2.966) |  | 1.579(1.005, 2.481) |  | 1.539 (0.946, 2.505) |  |
| >3.5 | 1.436 (0.905,2.278) |  | 1.129 (0.715, 1.783) |  | 1.031 (0.635, 1.672) |  |
| Missing | 4.750(2.138,10.550) |  | 3.923 (1.725, 8.923) |  | 3.804(1.600, 9.046) |  |
| Alcohol |  | 0.538 |  | 0.672 |  | 0.784 |
| No | 1.291 (0.658,2.533) |  | 1.091 (0.542, 2.197) |  | 1.007 (0.497, 2.037) |  |
| Yes | 1.985 (1.493, 2.639) |  | 1.545 (1.136, 2.100) |  | 1.264 (0.881, 1.814) |  |
| Missing | 1.942 (1.054,3.578) |  | 1.511 (0.832, 2.745) |  | 1.408 (0.678, 2.922) |  |
| Gout |  | 0.313 |  | 0.415 |  | 0.2900 |
| No | 1.846 (1.446, 2.357) |  | 1.480 (1.121, 1.953) |  | 1.274 (0.918, 1.769) |  |
| Yes | 1.081 (0.399, 2.923) |  | 0.950 (0.347, 2.601) |  | 0.709 (0.258, 1.951) |  |
| Cancer |  | 0.358 |  | 0.425 |  | 0.434 |
| No | 1.784 (1.342, 2.373) |  | 1.508 (1.117, 2.036) |  | 1.313 (0.919, 1.878) |  |
| Yes | 1.297 (0.723, 2.328) |  | 1.135(0.618, 2.084) |  | 0.973 (0.514, 1.841) |  |
| Smoke |  | 0.263 |  | 0.314 |  | 0.286 |
| Never | 1.611(1.125, 2.307) |  | 1.228 (0.835, 1.806) |  | 1.015 (0.671, 1.535) |  |
| Former | 2.436(1.536, 3.866) |  | 1.916 (1.183, 3.101) |  | 1.691 (0.980, 2.920) |  |
| Now | 1.446 (0.804, 2.601) |  | 1.251 (0.689, 2.272) |  | 1.129 (0.562, 2.267) |  |
| Stroke |  | 0.173 |  | 0.277 |  | 0.399 |
| No | 1.861 (1.457, 2.377) |  | 1.472(1.118, 1.939) |  | 1.261 (0.913, 1.743) |  |
| Yes | 0.866 (0.308, 2.440) |  | 0.794 (0.276, 2.284) |  | 0.762 (0.253, 2.294) |  |
| Vigorous activity |  | 0.239 |  | 0.173 |  | 0.439 |
| No | 1.683 (1.275, 2.223) |  | 1.277 (0.940, 1.735) |  | 1.155 (0.807, 1.652) |  |
| Yes | 2.356 (1.472, 3.769) |  | 1.887 (1.159, 3.072) |  | 1.465 (0.865, 2.481) |  |
| Moderate activity |  | 0.250 |  | 0.205 |  | 0.398 |
| No | 1.615 (1.213,2.149) |  | 1.250 (0.913, 1.712) |  | 1.117 (0.780, 1.602) |  |
| Yes | 2.162 (1.453,3.217) |  | 1.745 (1.141, 2.670) |  | 1.406 (0.882, 2.241) |  |

CI: confidence interval, OR: odds ratio

*Non-adjusted model adjusts for none. 
** Minimally adjusted model adjusts for age, gender, race. 
*** Fully adjusted model adjusts for age, gender, education, race, BMI, marital, PIR, gout, cancer, energy, HEI-2015, smoking, vigorous activity, moderate activity, alcohol, stroke.
